# Towards a Theory of Digital Well-Being: Reimagining Online Life After Lockdown

**DOI:** 10.1007/s11948-021-00307-8

**Published:** 2021-05-19

**Authors:** Matthew J. Dennis

**Affiliations:** Philosophy & Ethics Capacity Group, Eindhoven University of Technology, Eindhoven, The Netherlands

**Keywords:** Digital well-being, Social media technologies, COVID-19

## Abstract

Global lockdowns during the COVID-19 pandemic have offered many people first-hand experience of how their daily online activities threaten their digital well-being. This article begins by critically evaluating the current approaches to digital well-being offered by ethicists of technology, NGOs, and social media corporations. My aim is to explain why digital well-being needs to be reimagined within a new conceptual paradigm. After this, I lay the foundations for such an alternative approach, one that shows how current digital well-being initiatives can be designed in more insightful ways. This new conceptual framework aims to transform how philosophers of technology think about this topic, as well as offering social media corporations practical ways to design their technologies in ways that will improve the digital well-being of users.

## Introduction

The COVID-19 pandemic has caused a spike of concern about whether social media technologies (SMTs)^1^ undermine digital well-being (DWB).^2^ Since April 2020, large populations have spent their waking hours using SMTs to get news updates, communicate, and socialise online. ‘Doom scrolling’, ‘Zoom fatigue’, and what has become known as ‘digistraction’ are now everyday terms for many under extended lockdowns. This recent concern about DWB builds on a decade of growing public anxiety. Much of this has come from users of SMTs directly, although NGOs^3^ and law makers^4^ are now also taking a keen interest. On top of these recent worries, lockdown conditions caused by the pandemic have offered us a prescient glimpse of what our future relationship with SMTs might look like. The division between our online and offline lives is rapidly changing. Not only will SMTs will play an increasingly important role in our online lives, they will disrupt and reorganise our relationship between online and offline activities. Furthermore, there are signs the effects of the pandemic will be long-lasting. Many activities (teaching, working, socialising, etc.) are likely to adopt hybrid online-offline models after the pandemic has passed. This means that formulating a comprehensive theory of DWB has perhaps never been so important. So how does theoretical research on DWB, and practical initiatives aiming to promote it, fare under extended lockdowns? Do these conditions offer a useful lens through which to discern how SMTs could be better integrated into our daily activities? What are the alternatives to how this is currently done?

Theoretically speaking, ethicists are split on how to tackle DWB. Early in the debate, *value-sensitive design* (VSD) theorists argued that we can design for well-being in general (Brey, 2015, Brey et al., 2012; van de Poel, 2012; Swierstra & Waelbers, 2012; Oosterlaken, 2015; van den Hoven et al., 2017; Hoven et al., 2015), which inspired human–computer interaction (HCI) researchers to speculate how these techniques could be applied to DWB specifically (Calvo & Peters, 2013, 2014; Desmet & Pohlmeyer, 2013). More recently, some have argued that DWB is best promoted by *cultivating character traits* that align with SMTs to promote online flourishing. Shannon Vallor offers an account of ‘techno-moral virtues’ (2016), whereas Tom Harrison proposes a contrasting account of ‘cyber virtues’ (2016. Cf. Dennis & Harrison, 2020). Most recently, researchers from the Oxford Internet Institute (Burr et al., 2020a, b) have argued that the complexity of DWB requires a *multidisciplinary approach*, one that requires us to combine resources from empirical disciplines (psychology, sociology, STS), normative ones (ethics, law), as well as finding ways to practically apply these insights (design, engineering).

Practically speaking, few of these theoretical insights have been applied to the design of SMTs. In fact, I contend that the conceptual paradigm that the tech industry currently use to design for DWB is (1) insufficient and (2) urgently needs to be enriched with existing theoretical resources. DWB has been a trendy topic for social media companies (and related providers) since 2018. Twitter, Facebook and Instagram, Snapchat each launched a ‘digital well-being page’ for their users that year, just as Google’s Android and Apple’s iOS were overhauled in ways that these companies claimed would prioritise DWB. While each of these initiatives has merit, the first goal of this article is to show why thinking of DWB according to this corporate paradigm hobbles the radical approach which we need to reimagine DWB in a post-COVID world. This is especially troubling because this limited conception of DWB has deeply influenced the NGOs charged with restraining corporate tech and protecting consumers. NGOs such as the Center for Humane Technology (CHT), I contend, have adopted an invaginated version of the very same conception of DWB used by Google, Facebook, and their ilk. Identifying what both corporate and NGO approaches have in common is a key step in reimagining DWB in ways that move beyond existing approaches. By doing this, I suggest, can we employ the vital theoretical resources that those ethicists of technology listed above have developed over the last decades.

The aim of this article is to prepare the conceptual ground for a theory of DWB. A key aspect of this approach is to show what is currently missing, so I identify the common paradigm, one that is shared by both corporate initiatives (such as Google) and NGOs (such as the CHT). I call this common paradigm the ‘McDonald’s Model’ (McM) because both Google and the CHT view DWB in terms of moderation, self-regulation, and personal responsibility.^5^ This approach, I suggest, is reminiscent of how fast-food industry lobbied against regulations in the 1990s. Faced with imminent regulation, fast-food companies embarked on a mass marketing campaign to argue that their products were safe, *if they were consumed as part of a heathy lifestyle*. Because the consumption of fast-food was framed as a personal choice, it meant that providers were not liable for any detrimental health effects caused by the food they sold. The danger of current approaches to DWB is that they are following the McM, blaming users for problems in their DWB, while absolving the providers of SMTs of any responsibility. Understanding the weaknesses of a McM approach to DWB, I contend below, is the key to showing how we can reimagine flourishing online. In the final section, I sketch out what a reimagined conception of DWB that rejects the McM might look like.

## Recent Approaches to Digital Well-Being

So how do tech companies like Google understand DWB? And does this differ to how NGOs like the CHT understand it? I contend that both approaches view DWB in strikingly similar terms. I also argue that this conceptual similarity hampers our understanding of DWB because it makes unwarranted assumptions about how SMTs can be integrated into a flourishing life, while ignoring the manipulative effects of these technologies. To examine corporate initiatives, I focus on Google. This is because they has led other industry players (Facebook, Instagram, Twitter), but also because Google has made the key aspects of their approach to DWB publicly available. To examine NGOs, I explore the CHT, briefly comparing their method with that proposed by the American Academy of Pediatrics.

### Google’s Approach

Google officially launched their range of DWB solutions in 2018 at their annual developer conference, I/O. At this event, CEO Sundar Pichai claimed that Google’s existing products and services would be modified to reflect what he termed their four ‘Digital Well-Being Values’: (1) ‘Providing Awareness’, (2) ‘Enabling Control’, (3) ‘Delivering Benefits’, and (4) ‘Ensuring User Trust’. Designing Google’s technologies with these values in mind, Pichai proposed, would aim to give users ‘more control over their attention’ because ‘great tech should improve life, not distract from it’ (I/O 2018). While some philosophers would contest that 1–4 are ‘values’ in any substantive sense, they do represent a kind of evaluative commitment. It is also easy to see how they have guided the design of Google’s then newly launched operating system, Android Pie, along with its four DWB features: AppTimer (limits on app use), Shh (silencing of mobile device if orientated downwards), WindDown (greyscale in the hours before sleep) and Dashboard (overview of app use across devices). Regarding YouTube, Pichai announced an: (1) end to automatic continuous viewing, (2) a schedule a digest of notifications, (3) inserted break reminders, and (4) designated quiet hours (I/O 2018). These changes, Pichai claimed, reflect Google’s new vision for online experience that regards DWB as one of its three ‘chief principles’ (I/O 2018).

While grand ethical pronouncements by CEOs can sometimes be safely treated with scepticism, since Pichai’s announcement, Google’s drive to improve the DWB of its users has continued in important ways. In 2019, DWB was once again a major theme of I/O. In the ‘Improving Digital Well-Being’ break-out session, Rose La Prairie, Google’s newly appointed ‘Product Manager for Digital Well-Being’,^6^ shared details of a recent study on the DWB of 95,000 world-wide Google users. The study confirms that DWB is a major concern of users, with 1/3 of them reporting that they have used an in-house Google DWB technology in the past twelve months (2018–2019). Google claims that this study proves that its DWB features work effectively. In one metric, Prairie claims, app timers encouraged users to stick with their goals a staggering 97% of the time, and Winddown reduced night use of mobile devices by an impressive 27% (I/O 2019), although many with first-hand experience of these technologies (including the author) may be sceptical.

In the 2019 presentation, Prairie and Google’s DWB team make much of studies of ‘feature phone’ users in Japan (non-smart phones). These users reported greater feelings of control of their lives, less FOMO (fear missing out), and more JOMO (joy of missing out). By using to a less-sophisticated device, Google’s study claims, feature phone users experienced few of the problems that plague smart phone users (so-called ‘phantom cues’, repeated refreshing of apps, and FOMO). Nevertheless, feature phone users with active (especially urban-based) lifestyles reported some problems, the most important of which was that a smartphone is required for a host of everyday activities in today’s information-driven societies. From paying for groceries to navigating, many activities require or are strongly facilitated with a smartphone. The challenge, Prairie explains, is to mimic how feature phones operate by giving users options to safeguard the benefits of being online, while eliminating those things that reduce DWB, such as inessential notifications.^7^

In summary, all the features that Google introduced between 2018–2019 aim to promote better DWB by limiting the amount of time users spend time online. While these tools cover broad aspects of online use, the majority (AppTimer, Shh, WindDown, Dashboard, etc.) aim to promote DWB by allowing users to *moderate* their use of SMTs. Take AppTimer, for example. This function allows users to self-regulate how long they wish to spend on an app, then gently notifies them once this limit is reached. After this, users are given three options: to continue using the app for ‘one more minute’, ‘five more minutes’, or to ‘ignore limit’ entirely. Whatever option they choose, users are required to take personal responsibility for their continued use. Since the design of AppTimer is based on the idea that DWB must be pursued by (1) *moderating* one’s use of SMTs, by (2) *self-regulating* this use, and by (3) taking *personal responsibility* for it, the onus of responsibility is on the user. This means that users can be (4) *blamed* for hitting the ‘five more minutes’ or ‘ignore limit’ options, which raises the possibility of feeling (5) guilt and shame when their DWB is compromised. Most consequentially, as we will see later, this *absolves* providers of SMTs of responsibility for the DWB of their users. I move to show how these stages constitute the conceptual paradigm that guides Google’s approach to DWB, but first we must compare Google’s approach to that offered by the CHT. This initiative presents itself in opposition to tech companies such as Google, but it shares key elements of precisely the same conceptual paradigm.

### Center for Humane Technology’s Approach

During the COVID-19 pandemic, the CHT has risen in prominence, now overshadowing all equivalent NGOs.^8^ Many ethicists who work on DWB may well find the CHT’s mandate promising. Its website describes how the organisation is ‘dedicated to creating the conditions for radically reimagined twenty-first century digital infrastructure’ (CHT 2020). It proposes that such change will come from reforming the business model of ‘social media companies’ who threaten our ‘well-being’ by ‘profiting from outrage, confusion, addiction, and depression’ (CHT 2020). Doing this, the CHT suggests, will involve three ‘levers of change’, the most important of which is transforming the public attitude towards SMTs.^9^ For example, the CHT’s public programme proposes we can ‘build a healthier relationship with technology’ by (1) ‘deleting toxic apps’ (‘Snapchat, Instagram, TikTok’ are regarded as especially problematic), (2) ‘delaying giving children social media accounts or smartphones’, as well as (3) following self-imposed rules, such as ‘dedicated time device-free time’ (CHT 2020). Similarly, to Google’s approach to DWB, each of these suggestions views DWB as the ability to moderate one’s use of SMTs, enjoying the benefits while avoiding the worst effects. Through a process of moderation and self-regulation, the CHT proposes, DWB can be increased.

The CHT’s guidance on DWB during lockdown exemplifies this logic of moderation and self-regulation. In April 2020, the CHT issued eight ‘Digital Well-Being Guidelines During the COVID-19 Pandemic’.^10^ These guidelines emphasise how self-regulation can be used to moderate one’s use of SMTs (CHT 2020; see Dennis, 2020b for detailed criticisms). The first guideline, for example, implores users to (1) ‘*make* a time management plan’ for online and offline activities, (2) ‘*reflect* on how [social media are] working with your well-being’, and (3) ‘*choose* digital environments that are supportive of the […] values you’re striving to live by’ (CHT 2020; emphasis added). The aim of following these guidelines is to *moderate* one’s use of SMTs, and 1–3 suggests that this can be done by using faculties that require *self-regulation*.

The CHT is not the only NGO to conceive of DWB as involving moderation and self-regulation. In May 2020, The American Academy of Pediatrics (AAP) launched a similar initiative to tackle the DWB of children and young people who had been forced to remain home during lockdowns, often only with distracted parents for company. To do this, the AAP produced a ‘Personalized Family Media Use Plan’ that encourages users to allocated reflection to how one is ‘using media to achieve your purpose’ and requires ‘parents and users to think about what they want those purposes to be.’ (AAP 2020) This can be done, the AAP suggests, by using their tool to ‘think about media and create goals and rules that are in line with your family’s values.’ The tool allows users to pledge to moderate their use of SMTs by self-regulating their online behaviour in conformity with the guidelines.^11^ From this we can see that the approaches of the AAP and the CHT have important commonalities. Most obviously, they assume that DWB involves users moderating their online/offline activities. In addition to this, both sets of guidelines presuppose a strong sense of personal responsibility—the onus of responsibility for DWB is placed on the consumer. Each of us is individually responsible for our own DWB, so we are encouraged to take it as an object of personal concern, to strive to maintain and improve it, etc. Apportioning personal responsibility has other consequences. If we are responsible for our own DWB, then we can be blamed when it goes awry, and may even be subject to guilt or shame. From this we can see that both the providers of online services and those who lobby for showing a greater role in DWB both adopt essentially the same paradigm. All these innovations have some value, but the assumptions of this overall conceptual paradigm remains unchallenged. I explain why this is a problem later in this article, but first it is important to explain precisely what this conceptual paradigm is.^12^

## What is the McDonald’s Model?

We have seen that there are currently two prominent approaches to DWB, one corporate and one belonging to NGOs seeking to promote the DWB of users. Prima facie both Google’s and the CHT’s approaches are dissimilar, even antagonistic. On the one hand, Google suggests that its products are harmless, as long as they are used as part of a healthy online life. On the other, CHT views SMTs as dangerous, so offers advice on how users can moderate their use of these technologies, one requiring self-regulation. Despite these surface differences, however, there are strong reasons to think that they are part of precisely the same conceptual paradigm. Identifying—and ultimately dismissing—this conceptual paradigm is an essential step in reimagining DWB. So what precisely does this conceptual paradigm consist in? In what sense are corporate approaches to DWB and those of NGOs similar? In what follows, I propose that we can answer both questions by showing how existing approaches promote what I call the ‘McDonald’s Model’ to DWB.

As mentioned in above, the McM takes its name from the regulative model that major fast-food chains adopted after lobbying policy makers to avoid legal regulation. Following growing scientific evidence that fast-food has serious health effects, customer-interest groups and NGOs advocated for greater industry regulation, including potentially banning many popular (and highly profitable) fast-foods. To counter this, companies argued that their products could be safely consumed as long as users did so as part of a healthy lifestyle. Faced with the possibility of imminent legislation that would have required fast-food companies to drastically reduce portions, cut unhealthy ingredients, and would have even outlawed some dishes, the industry embarked on an advertising campaign. In the face overwhelming evidence, industry strategists conceded that there were serious health problems with the regular consumption of fast-food, but that such adverse effects could be avoided by eating their products in moderation. This had massive benefits. For example, it allowed the fast-food industry to avoid the bans on TV advertising that had previously been imposed on the cigarette and alcohol industries. Most importantly, it prevented regulators from forcing fast-food companies to change their menus. Instead of legislating about the ingredients and portions of fast-food, the burden of responsibility was placed on the consumer. Rather than making outlets responsible for what they served, it made consumers responsible for what they ate. Consumers could enjoy a meal comprising of a burger, fries, and soda (or similar variations), as long as they balanced this with other foods and activities (sport, salads, etc.) This meant that obesity, heart disease, and tooth decay were reframed as problems that consumers had caused themselves because they had failed to exercise the required moderation in their consumption of the foods the industry offered. Current initiatives to DWB follow this model in two ways: first, they concede that the use of SMTs can be detrimental when not moderated as part of a healthy online/offline balance; second, they propose that is should be tackled by consumer behaviour (proposing that these problems can be avoided by self-regulation).

Table 1 compares the argumentative steps that both fast-food and SMT providers use to claim their products are safe in moderation. In the case of social media, the model claims that SMTs have no damaging effects as long as they are used in *moderation* (breaks from screentime). The model then proposes that (1) *moderation* can be achieved though (2) *self-regulation*, which leads to a view about (3) *personal-responsibility*. This has significant consequences. First, it allows users to be (4) *blamed* when their DWB is inadequate. Second, it conceptually underwrites feelings of (5) *guilt* and *shame* that blame can generate. Because users are responsible, they are regarded as blameworthy; because they are regarded as blameworthy they are subject to feelings of guilt and shame, even by those who are charged with helping them such as the CHT. The effect of this is that social media companies are (6) *absolved* from blame. Since the responsibility for DWB falls on the shoulders of users, then tech companies can deny that they are responsible for how their products are used. Just as the McM absolves fast-food corporations from the health effects of their products (obesity, cardiac problems, etc.), social media companies are free to offer users whatever content grabs their attention while having no responsibility for how this effects the user’s DWB. The (7) *upshot* is that social media companies are freed from the responsibility of offering products and services that are compatible with DWB.

**Table 1:** The McDonalds Model (McM): regulation of fast-food and social media technologies

	Key features	McM (fast-food)	McM (SMTs)
1	Moderation	Fast-food can be safely enjoyed as part of a *healthy overall lifestyle* (exercise and a varied diet)	SMTs can be safely enjoyed providing it is done in *moderation* (variety of other online and offline activities)
2	Self-regulation	To moderate consumption, fast-food diners can and should regulate *their own* consumption	To moderate consumption, online users can and should regulate *their own* use of SMTs
3	Personal responsibility	The burden of responsibility is on diners. They are *personally responsible* for their fast-food consumption	The burden of responsibility is on users. They are *personally responsible* for their use of SMTs
4	Blame	Fast-food diners are responsible for overconsumption, so they *can be blamed* for the health effects that fast-food causes (obesity, tooth-decay, etc.)	SMT users are responsible for excessive use of SMTs, so they *can be blamed* for effects this has on their digital well-being
5	Guilt/shame	Fast-food diners can be blamed for excessive fast-food consumption, so they *should feel guilt/shame* when they over consume	SMT users can be blamed for detrimental effects of SMTs on their digital well-being, so they *should feel guilt/shame* when they over consume
6	Absolution	Any detrimental effects from fast-food are the *diners’ fault*, not the fault of the fast-food provider	Any detrimental effects from online social media are the *users’ fault*, not the fault of social media companies
7	Upshot	Fast-food providers can *continue to sell, advertise,* and *promote* their products. They do *not need regulation*. They are *not liable* for any detrimental health effects of fast-food	Tech companies can continue to *sell, advertise,* and *promote* their services. They do not need to be regulated. They are *not liable* for detrimental effects of SMTs on digital well-being

## Problems with the McDonald’s Model

Now that I have identified the key features of the McM, I am ready to evaluate it. The main objections to applying the McM to SMTs are that this model (1) *harms* users, (2) it fails to account for the *manipulative nature* of SMTs, which (3) means that this model is *disingenuous*. Taken together, these objections indicate that an adequate account of the relation between SMTs and DWT requires an alternative conceptual paradigm. I sketch the key challenges for such a future paradigm in the final section.

### Harm

There is increasing evidence that SMTs can *harm* the DWB of users (Goh et al., 2019; Goodyear et al., 2018; Samad et al., 2019; Twenge et al., 2020), although the extent of these harms are disputed.^13^ Since choosing the right regulatory framework depends on the potential harms of the product or activity that the framework is regulating, if SMTs negatively impact on DWB, then they should be regulated with a framework that reflects the dangers of SMTs. This can be illustrated using two comparative examples. The McM is *unobjectionable* in domains where it regulates non-harmful products or service, for instance in regulating physical exercise. Gym-goers are well advised to employ the concepts of ‘moderation’, ‘self-regulation’, and ‘personal responsibility’ in their approach to exercise, as can personal trainers and providers of gym equipment. While we should strive to avoid pernicious kinds of ‘blame’ (such as body shaming or perfectionism, etc.), it is okay that the McM applied to exercise would encourage guilt about missing an exercise session or shame if a gym-goer lets their exercise partner down. Nevertheless, it would be *objectionable* to apply the McM to products and services that are explicitly harmful, such as cigarette smoking. As medical evidence against smoking mounted in the 1960s, the tobacco industry was unable to deny the risks of smoking. Legislation meant that this industry was forced to move from a conceptual paradigm of ‘self-regulation’ to one based on ‘self-imposed risk’. Today, smokers are granted the right to smoke, but there is no legal provision for them to blame (or sue) cigarette manufacturers. Instead, regulative bodies attempt to discourage self-imposed risk of smoking with repellant cigarette packaging and negative advertising campaigns.

Unlike smoking, the evidence of the harm of SMTs are currently disputed. This is why comparing SMTs to fast-food is more informative. Compared to smoking, both the harms and addictive qualities of fast-food and SMTs are fiercely contested. Nevertheless, in recent years an increasing number of studies have shown that fast-food is both addictive and harmful (even in small qualities). Documentaries such as *Super-Size Me*, have drawn public attention to the dangers of fast-food for health, just as *The Social Dilemma* has drawn attention to the danger of SMTs for DWB. The McM was proposed by fast-food companies in the midst of ambiguous evidence of their harms, so too precisely the same model is now being invoked by social media companies, who are both at a state of epistemic uncertainty regarding their potential dangers and are currently striving to avoid stringent regulation. If theoretical concern regarding these dangers is justified, then we must think again about the conceptual paradigm that underwrites the regulatory framework we use.

### Manipulation

Recently SMTs have been shown to be highly effective at *manipulating* user behaviour (Frank, 2020; Ham & Spahn, 2015; IJsselsteijn et al., 2006; Lanzig, 2019). While SMTs do this in multiple ways,^14^ manipulation creates a direct problem for the McM if SMTs turn out to be addictive. In fact, this is precisely what researchers have shown. Many SMTs incorporate persuasive technologies that are extremely effective at keeping us hooked online, which has even led some theorists to describe these technologies as *addictive.* The McM only makes sense when our faculties of autonomy and free choice are relatively unrestricted, as these faculties provide the conditions for self-regulation and personal responsibility. If SMTs have features that significantly curtail these faculties, then they must be regulated by a conceptual paradigm that is compatible with autonomy and free choice. Many smokers will agree that self-regulation is an ineffective way to regulate their consumption of cigarettes. It is ineffective because the addictive qualities of tobacco undercuts the ability of smokers to self-regulate. This means that it makes little sense to blame an addicted smoker for their habit, nor would it be fair to act in ways that cause the smoker feel guilt or shame about it. If SMTs are addictive, it will be hard to moderate our use of them as this depends on our capacity for self-regulation, which persuasive technologies have been shown to undermine. This means that it makes little sense to blame users when they use SMTs in ways that are detrimental to their DWB, if SMTs include manipulative technologies to keep users hooked to their services.

Whether the addictive qualities of SMTs are more similar to fast-food or to cigarettes is currently an open question. As we have seen, whereas the addictive potential of fast-food is disputed, the addictive nature of smoking is indisputable. SMTs have not been shown to cause physical addiction (akin to the physiological craving for nicotine), but there is growing evidence that they can be responsible for psychological addiction (in a comparable sense to how persons can be addicted to gambling). Currently, the evidence concerning the addictive nature of SMTs is mixed (Grau et al., 2019; D’Arienzo et al., 2019), but some users clearly find them addictive. This indicates that we have reason to think of them more like cigarettes, rather than fast-food. Just as we accept that different regulatory models should be applied to fast-food than to cigarettes, we should worry whether the McM is the best ways to regulate our use of SMTs. We can only moderate our use of SMTs, if these technologies are not addictive to the point that they compromise our capacity to self-regulate our use of them.

### Disingenuousness

I have proposed that there are strong reasons to think that SMTs (1) cause harm, and (2) are manipulative. If there are reasons to think this, then applying the McM to SMTs is *disingenuous*. To propose that the users of SMTs moderate their use of these technologies through self-regulation could only be a viable approach if SMTs did not undermine the very conditions under which self-regulation could take place. If SMTs are significantly addictive (even in a minority of users), then it would be disingenuous to base the rules governing them on a conceptual paradigm that presupposes that moderation is possible. If the McM cannot apply to the regulation of addictive entities, then proposing it as the correct conceptual paradigm to understand SMTs offers users false hope in using these technologies in ways that are compatible with their DWB.

The charge of disingenuousness is compounded if we consider some of the broader consequences of the McM. As Table 1 shows, aiming for moderation via self-regulation provides the conceptual possibility for thinking of DWB as a personal responsibility. This places users on a slippery slope. Failing to moderate their consumption of SMTs, opens users to blame if their DWB lapses. Understanding DWB as something that we can be blamed for causes further damage. It underwrites the possibility of social opprobrium. Users of SMTs are accountable for their own DWB, and can be blamed if it is not adequate. Such blaming leads to guilt or shame about insufficient DWB. Starting out from understanding DWB as the moderate use of SMTs, ultimately provides the conditions for blaming the user if their use is not moderate.

From this we can say that applying the McM to SMTs seems disingenuousness because it blames users for their immoderate use of addictive SMTs. The upshot of this is that the McM absolves social media technology companies (Twitter, Facebook, Snapchat, TikTok) and the providers of digital infrastructures (Google, Microsoft). While it is beyond the parameters of this article to speculate on the motives of executives for adopting such a model, it is perhaps unsurprising that they have opted for an approach that saddles users with personal responsibility for their DWB, and absolves themselves. Yet, given what I have said about how SMTs can be manipulative, and can even cause addiction, such approach is disingenuous because those advocating the McM know that it is practically impossible to sustain.

## Beyond the McDonald’s Model

In the previous section I argued that the problems with applying the McM to SMTs are formidable. So, if we relinquish this conceptual paradigm, what kind of framework would be a suitable replacement? Answering this question, I contend, requires us to think about alternative ways to regulate our use of SMTs. Practical initiatives to improve DWB need to move beyond the McM. To do this, I propose, they need to base themselves on a conceptual paradigm that avoids the pitfalls listed above, as well as facing the following challenges:

### Beyond Moderation (Online/Offline Distinction)

As we have seen, moderation is the grounding principle of the McM. Social media companies propose that digital life should include a healthy balance of online and offline activities. On the one hand, SMTs are presented as offering highly efficient ways to connect with friends, discover new things to do, and share our lives with one another. On the other, social media companies propose that DWB requires spending time offline too (*spending time* with friends, *doing* new activities, and actually *accomplishing* whatever we use SMTs to share). This means that the problem is twofold. First, an increasing number of everyday activities involve logging on, often with a smart device, so thinking of DWB as a healthy balance of online and offline activities is often practically unsustainable. In today’s information-driven societies, accomplishing everyday tasks without online access can be hard. Despite Google’s efforts to replicate the benefits of so-called ‘dumb phones’ by offering options to limit smart-phone functionality, social media companies are increasingly designing their platforms so that navigating (via Google Maps), everyday communication (via Facebook Messenger), and even listening to music (iTunes) require us to be online and logged with a SMT user profile.^15^ Second, there are strong indications that this will increase in the post-COVID world. Since March 2020, SMTs have become increasingly popular to communicate, socialise, and play games. Simultaneously, other online technologies (i.e. video-calling) have become integrated with SMTs, so that we can integrate the benefits of these technologies in conference calls, online education, and when receiving medical advice.

There are two ways to face these challenges. The most obvious is to resist situating ever-more ordinary activities online, especially when doing so offers little benefit to users. For example, while a laundry company may benefit from creating an app so that customers do not require loose change, this will require customers to engage in a series of complex online activities (downloading the app, uploading credit, etc.) Not only may this be time consuming, but it requires that users are continuously connected. This is an argument for situating activities offline, so that there are natural spaces and gaps in online activity of users. Regarding essential services that have been situated online due to COVID-19 (education, medical care, etc.), they should revert to offline equivalents as soon as possible. Nevertheless, there are signs that COVID-19 will present long-term challenges for DWB, many of which will not disappear once the pandemic has passed. SMTs are showing signs of becoming more-and-more embedded in essential services, so may well be permanently incorporated into communication, education, health, and leisure even once the virus is under control. This means that we cannot only rely on arranging our practical lives in ways that make space for offline activities. Paradigmatic changes that offer ways to design for DWB that are not based on the McM will also be required.

One paradigmatic change would be stop thinking of DWB as immoderate amounts of screentime. Today, SMTs are overwhelmingly screen-based activities, but we could rethink how to design these technologies in ways that do not require their users to face a screen. In fact, screentime is closely associated with the *moderation* of the McM, as moderation is invariably understood as *moderation of the time one spends staring at a screen*. As we have seen, this creates the tension with contemporary ways of living that require us to be online continuously, a tension that has been exacerbated by DWB problems connected to lockdowns (Zoom fatigue, digistraction, doom-scrolling, etc.) Despite this, if extended screentime is a threat to DWB, and lockdown conditions require us to use screen-based SMTs for longer, one simple design recommendation could help. Designing SMTs in ways that are more audio-based would undercut the idea that DWB should be thought of in terms of a balance between being online and offline. This could allow users to be online for extended periods, albeit in a way that was less deleterious to their DWB because being connected would be a ‘background’ activity. Video- and image-communication are currently prioritised on the most popular SMTs (Facebook, Instagram, Snapchat), so prioritising voice communication would be one way to challenge the offline/online distinction of the McM, while also allowing users to enjoy the benefits of being online. If being continually connected to the Internet is inescapable, then designing SMTs so they can be used unobtrusively eschews the need for us to schedule regular times to take a break.^16^

### Beyond Self-Regulation

We have seen that self-regulation is the means through which the McM proposes to moderate our use of SMTs for the purpose of improving DWB. Earlier we saw that self-regulation is how both Google and the CHT frame DWB, either with technological innovations to curb our unrestricted use of SMTs (Google) or by offering us non-technological tools of self-regulation SMTs (CHT). Yet, all too often, many find themselves chronically distracted, doom scrolling, and spending much more time using SMTs than they originally intended. Take, for example, trying to curtain online use by using YouTube’s ‘Take a Break’ function. Although a user may have resolved to only spend, say, twenty minutes viewing each day, many will be familiar with the phenomenon of continually overriding this option, opting for ‘5 more minutes’ or ‘ignore limit’ when notified that they have exceeded their self-imposed viewing limit. How might an alternative conceptual paradigm support other ways of curtailing the damaging effects of SMTs?

One possibility is that the resources to answer to this question could be found in theoretical accounts of DWB. When exploring these accounts above, I noted that VSD theorists have already shown that it is possible to design artifacts for well-being in general, and that HCI researchers have indicated that this might be applied to DWB specifically. Unfortunately, few of these initiatives have received uptake in Silicon Valley, due to the dominance of the McM. Instead, the providers of SMTs claim that the McM can solve DWB, which offers them a rationale to reject the idea that more systemic solutions are needed to tackle this problem. Indeed, I have suggested that NGOs such as the CHT are right to say that social mediascompanies are not motivated design these technologies with the aims of DWB. Rather, as the CHT claims, social media providers typically have more mercantile motivations, such as increasing the screentime of their users for the purposes of advertising revenue or for data extraction.

Furthermore, the evidence adduced above suggests another reason why the McM is not an adequate way to think of DWB. Self-regulation cannot work as effectively as the proponents of this conceptual paradigm claim because SMTs are riddled with persuasive technologies that are explicitly designed to undermine our ability to regulate our time online. From a practical point of view, self-regulation is impossible for many of us, which led to my charge of disingenuousness. This means that applying the insights of VSD to SMTs has much potential. VSD theorists have shown that values can be successfully embedded into artefacts, and HCI researchers have shown that in principle this can applied to the design of online architecture. The hope is that, by taking the DWB more seriously, the providers of SMTs can design online environments that prioritise the value of DWB and offer ways to practically promote it. Google’s four ‘Digital Well-Being Values’ are a welcome first step, but a more philosophically robust set of values would to enable designers to create SMTs that more fully reflect the value of online human flourishing.

### Beyond Personal Responsibility (and Blame, Guilt, Shame)

Personal responsibility is connected to self-regulation. If we are required to self-regulate our use of SMTs to promote our own DWB, then we have personal responsibility for any problems we encounter when doing this. While personal responsibility can be appropriate in some contexts, it is typically inappropriate in contexts where we have few or seriously diminished choices. The problem is compounded once we recognise that we can be blamed, if we are regarded as failing to meet our personal responsibilities. Blame provides the conditions for both guilt and shame, which is especially unfair if we consider the manipulative qualities of some SMTs. We have seen above that SMTs use a range of persuasive technologies to manipulate us into staying online for extended periods. This means that we can be blamed for something (and it is implied we should feel guilt and shame about it) when moderating the use of SMTs is beyond the volition of most users.

The current manipulative power of online environments suggests that the best way to solve this problem is by designing SMTs in ways that they do not require self-regulation. The theoretical methods of VSD theorists and practical tools of HCI researchers suggests that this is possible in principle, but it is important to note that these possibilities are at odds with how social media companies currently design their platforms. The CHT is right to say that today’s SMT companies invariably design online architecture that aims to keep us scrolling and clicking to the point of practical addiction. This indicates that SMT companies need to actively design for DWB, using the principles developed by VSD theorists. Yet the motivation for this cannot come from users. Systematic changes will have to be legislated for, so the CHT is right to supplement its central mission of ‘educating the public’ with two ancillary aims: (1) ‘Informing Policy Change’ and (2) ‘Supporting Technologists’.

Despite the importance of systemically changing the design of SMTs, we should not give up on the idea that moderation, self-regulation, and personal responsibility have a role to play in maintaining DWB. There is little sign that the insights of VSD theorists will be applied in the near future. This means that user-focused ways to improve DWB that do not require a systemic approach should continue to be supported, especially while the pandemic continues. I have questioned whether the CHT’s rule-based approach will be effective in the face of the powerful persuasive technologies of SMTs, but the idea of inculcating character traits that promote DWB may hold more promise. Empirical work on the effectiveness of character-based approaches suggests that they give rise to changes in behaviours that can be more deep seated than simply aiming to follow the rules. Whether the ‘techno-moral virtues’ of Vallor, or the ‘cyber-virtues’ of Harrison, would be most effective is an open question, but there are reasons why instilling deep-level character traits that helps regulate online activity may be more effective than setting personal screentime limits. While the psychological studies invoked by much of the situationist literature shows that character traits are often brittle in the face of environmental stimuli, moral psychologists responded character traits still play an significant role, albeit a less that important one that has often been assumed by virtue ethicists (Alfano, 2013).

In combination with a VSD approach to building online architecture that actively supports DWB, a character-based approach to DWB may have much to offer. On the one hand, it is clear that much of our online behaviour can be explained by environmental stimuli, which is clearly shown when we consider how effective the persuasive aspects of SMTs can be. On the other, this is not the whole story. There is now significant empirical evidence that a character-based approach can explain human behaviour to some extent (Alfano, 2013. Cf. Doris, 1998, 2002). While this means that there is some room for personal responsibility, this need not extend to blaming ourselves or feeling guilt or shame when our DWB is undermined by the persuasive technologies that social media companies employ.

### Beyond Corporate Absolution

The McM proposes that DWB is moderating one’s use of SMTs though self-regulation, so companies that adopt this model are effectively given an alibi. Creating DWB tools that depend on the ability of individuals to self-regulate their online consumption, allows SMT platforms to *appear* to respond to customer concerns regarding DWB, while not making systemic changes to their services. Just as the McM absolves the purveyors of fast-food of the responsibility to change their recipes, promoting DWB according to a conceptually similar model absolves social media companies from their own responsibilities. We have already touched on the antidote to this above (5.3). Thinking of DWB beyond personal responsibility, requires that SMT companies think about the design decisions that implicitly structure their online architecture. Specifically, this means that SMT companies must reflectively decide on the values for which they wish to design. Instead of concentrating on ways to design online architecture that maximise user engagement, for example, companies could think more creatively about designing for the DWB of users, even if this may well simultaneously require taking into account values relating to the amount of time users spend online. Whether such a potential value conflict could be successfully negotiated or not, VSD provides a promising way for SMT companies to take on the responsibility for the DWB of their users. Moreover, this is an opportunity that SMT companies would do well to take. Given what we saw about the disingenuousness of promoting DWB in terms of personal responsibility, at the same time as actively enlisting manipulative technologies that undermine personal responsibility, VSD offers SMT companies a way to take the responsibility for the DWB of their users while avoiding the problems of the McM that I set out above. Viewing DWB as a responsibility that individuals and corporations both share, dispenses with the myth that SMT companies can use the McM to absolve themselves of responsibility for the DWB of users.

## Conclusion: Reimagining Digital Well-Being in a Post-Pandemic World

The emergency lockdown measures required to contain COVID-19 provide a unique viewpoint on a future in which SMTs play an increasing role in our lives, as well as alerting us to how SMTs can impact upon DWB. Under lockdown conditions, many now use online technology from the moment we wake to the time we go to bed, there is good reason to attend to think critically about the conceptual understanding of DWB with which we develop future SMTs. Corporate providers of SMTs (Twitter, Facebook, Snapchat, etc.) and those who provide underlying services (Google, Microsoft, etc.) adopt an approach to DWB that I have termed the McM, an approach that is followed by NGOs charged with protecting users (CHT, AAP, etc.) While the CHT’s ‘Digital Well-Being Guideless During the COVID-19 Pandemic’ may be useful for some users, relying them on underestimates the manipulative power of SMTs to keep users continuously online. Furthermore, the McM saddles users with the complete responsibility for their online behaviour, letting the providers of SMTs off the hook. It is understandable (albeit regrettable and disingenuous) that tech companies would be happy with the McM, but it is less understandable why NGOs charged with safeguarding the DWB of users should adopt the same conceptual paradigm.

Looking ahead, the way we organise our lives when faced with a global pandemic may provide clues to how we can respond to future ecological challenges. Three years prior to the pandemic, Vallor cautions that in the future we may be required to face ‘environmental degradation’, ‘resource depletion’, and ‘global economic and climate instability’ (2016: 117). While Vallor was surely thinking of far-flung future problems, at least some of these challenges could be mitigated by travelling less, home working, etc. It is easy to imagine how responding to such challenges might involve living under lockdown-like conditions, relying heavily on SMTs to work, communicate, socialise, seek medical information, etc. Understanding the inadequacy of current approaches to DWB, should encourage us to pursue a comprehensive theory of living well online that can be practically applied to help us face the challenges of the future. SMTs could have a fundamental role to play in maintaining a distinctively human life when confronted with a variety of difficulties. Understanding how we can make use of these advantages while still maintaining DWB, is a challenge that the COVID-19 pandemic offers the opportunity to face.

## Footnotes

1 I use the term SMTs to designate both social media platforms such as Facebook, Instagram, Snapchat, and Twitter, and the providers of software and hardware that these platforms use to offer their services (Google, Microsoft, etc.).

2 I follow Christopher Burr and Luciano Floridi’s definition of DWB as: the ‘impact that digital technologies, such as social media, smartphones, and AI, have had on our well-being and our self-understanding of what it means to live a life that is *good for* us in an increasingly digital society.’ (Burr & Floridi 2020: 3; emphasis in original. Cf. Burr et al., 2020b: 1).

3 For an overview of the activities of Center for Humane Technology (CHT), see Dennis (2020a).

4 US Congress Subcommittee: ‘Americans at Risk: Manipulation and Deception in the Digital Age’ (8th January 2020).

5 I use the term ‘McM’ in an alternative sense to George Ritzer’s term ‘McDonaldization’ (1992), which he uses to describe the ‘efficiency, calculability, predictability, and control’ of current business models (1992: 20). Thanks to an anonymous reviewer for this reference.

6 Rose La Prairie. www.youtube.com/watch?v=8dH7gmpF5WQ.

7 To combat user distractions, for example, Gmail launched a ‘Priority’ notification system (reducing email notifications by up to 97%), a ‘Scheduled Send’ function (allowing users to be more thoughtful about when emails arrived), and a ‘Focus’ mode (pausing notifications entirely).

8 Most prominently, the CHT features heavily in *The Social Dilemma* (2020), a Netflix documentary about its work. See Dennis (2020b) for a critical review.

9 The CHT calls its central task: ‘Educating the Public’, but it is complemented by ‘Informing Policy Change’ (see Tristan Harris’ remarks at the Congressional hearing), ‘Supporting Technologists’ (see CHT’s tech developer guidelines).

10 ‘Digital Well-Being Guidelines During the COVID-19 Pandemic.’ Available at: www.humanetech.com/digital-wellbeing-covid [Accessed September 30th 2020].

11 See AAP website: www.healthychildren.org/English/media/Pages/default.aspx (accessed September 2020).

12 While it is unsurprising that the assumptions that corporate tech makes about DWB are compatible with using its products, it is odd that NGOs such as the CHT and AAP do so too. Perhaps this is indicative of the fact that many of the key players from at least the CHT are Silicon Valley insiders, often boasting resumes of their prominent positions with companies such as Google and YouTube, Facebook and Instagram, etc.

13 See Orben & Przybylski (2019a, b). Most research on SMTs has focused on how they cause us to spend extended periods of screentime, which is viewed as detrimental to DWB (Burr & Floridi, 2020; Burr et al., 2018; Klenk, 2020; Lanzig, 2018, 2019; Sullivan & Reiner, 2019). See Dennis (2020a) for an account of how we can think DWB beyond screentime.

14 Philosophers have convincingly argued that manipulation is in tension with well-being in general (Alexandrova, 2017; Bishop, 2015; Fletcher, 2015, 2016) because it undermines paradigmatic human faculties (deliberation, choice, etc.) and related values (responsibility, autonomy). If SMTs are manipulative, then this is in tension with DWB because it interfers with the exercise of these fundamenal faculties along with these values.

15 Examples are manifold. To use bluetooth headphones, one must be online, which means that one must receive notifications from social media accounts, which are now designated as ‘read’ and awaiting a response.

16 While substituting visual for audio-based services may mitigate challenges for DWB that concern screentime, audio-based services cannot be regarded as a panacea. Following Burr & Floridi’s definition (2020; cited in footnote 2), DWB concerns our *entire* relationship to online activities. Audio-based online activities can be extremely compulsive—as many podcast listeners can attest. It is perhaps no accident that since the pandemic began there has been a boom in providers of these services (e.g. Clubhouse). I would like to thank an anonymous reviewer for drawing my attention to this.
